# School-based Programs: Lessons Learned from CATCH, Planet Health, and Not-On-Tobacco

**Published:** 2007-03-15

**Authors:** Adele L Franks, Steven H Kelder, Geri A Dino, Kimberly A Horn, Steven L Gortmaker, Jean L Wiecha, Eduardo J Simoes

**Affiliations:** Prevention Research Centers Office; Center for Health Promotion and Prevention Research, University of Texas Health Science Center at Houston, Houston, Tex; Prevention Research Center, Robert C. Byrd Health Sciences Center, West Virginia University, Morgantown, WVa; Prevention Research Center, Robert C. Byrd Health Sciences Center, West Virginia University, Morgantown, WVa; Prevention Research Center on Nutrition and Physical Activity, Harvard University, Cambridge, Mass; Prevention Research Center on Nutrition and Physical Activity, Harvard University, Cambridge, Mass; Prevention Research Centers Program, CDC, Atlanta, Ga

## Abstract

Establishing healthy habits in youth can help prevent many chronic health problems later in life that are attributable to unhealthy eating, sedentary lifestyle, and overweight. For this reason, many public health professionals are interested in working with school systems to reach children in school settings. However, a lack of familiarity with how schools operate can be a substantial impediment to developing effective partnerships with schools.

We describe lessons learned from three successful school health promotion programs that were developed and disseminated through collaborations between public health professionals, academic institutions, and school personnel. The programs include two focused on physical activity and good nutrition for elementary and middle school children — Coordinated Approach to Child Health (CATCH) and Planet Health — and one focused on smoking cessation among adolescents — Not-On-Tobacco (N-O-T).

Important features of these school health programs include 1) identification of staff and resources required for program implementation and dissemination; 2) involvement of stakeholders (e.g., teachers, students, other school personnel, parents, nonprofit organizations, professional organizations) during all phases of program development and dissemination; 3) planning for dissemination of programs early in the development and testing process; and 4) rigorous evaluation of interventions to determine their effectiveness. The authors provide advice based on lessons learned from these programs to those who wish to work with young people in schools.

## Introduction

Schools can play a crucial role in improving the health of children and, thus, the adults they will become. Children and adolescents generally attend school 5 days per week throughout most of the calendar year. Schools in the United States are located in communities of every socioeconomic, racial, and ethnic group. In addition to academic skills, students also learn cultural expectations and social norms that strongly influence health behaviors (1).

During childhood and adolescence, habits emerge that influence and reinforce physical activity, eating, and tobacco-use behaviors. These health behaviors can affect development of cardiovascular disease, cancer, and diabetes, which are now the major causes of premature death and disability in the United States and the Western world (2). Public health professionals are interested in school-based programs that can provide a foundation for lifelong healthy behaviors and thereby significantly reduce the burden of preventable chronic diseases for both individuals and society.

Although more than 80% of U.S. school districts require health education to be taught in their elementary, middle, and high schools, less than half provide a curriculum for teachers (3). Furthermore, available curricula have rarely undergone rigorous evaluation to demonstrate their effectiveness, and they are often out-of-date when compared with current practice standards (4). Thus, there is a largely unmet need for effective school-based programs that promote healthy behaviors among youth. For public health officials who have embraced the concept of evidence-based practice (5), it is imperative that programs be rigorously evaluated. Collaboration between the public health and education sectors is essential to ensure that effective programs are available and meet the needs of youths, teachers, and school administrators.

Creating public health partnerships with schools is challenging for many reasons, including the numerous academic and nonacademic demands placed on schools. In addition, school programs often lack sufficient funds, are subject to political vicissitudes, exist in complex bureaucracies that foster fragmentation, and vary across localities (6). Despite these problems public health and education sectors have worked together successfully on programs that can serve as examples to guide and encourage future collaborative efforts.

The Division of Adolescent and School Health at Centers for Disease Control and Prevention (CDC) funds collaborations between state education and health agencies to promote coordination of school health programs. CDC's Prevention Research Centers (PRCs) engage public health organizations, academic institutions, and communities in partnerships to develop, test, and disseminate programs to improve health outcomes. To be considered effective, these programs must undergo systematic measurement and analysis using solid research methods and study designs. In some cases a PRC participates in the development and testing of new programs; in other cases a PRC participates by studying how to disseminate programs already shown to be effective. This paper explores three effective school-based programs promoted by PRCs and highlights common features and lessons learned for public health practitioners and researchers who wish to partner with schools. Two of these programs promote physical activity and healthy eating among school children — Coordinated Approach to Child Health (CATCH) and Planet Health — and one promotes smoking cessation among  adolescents — Not-On-Tobacco (N-O-T).

## Coordinated Approach to Child Health (CATCH)

CATCH is an elementary school program focused on supporting positive environmental influences to increase physical activity and improve healthy eating. CATCH involves classroom and physical education teachers, school food service personnel, and families of students. Input from school administrators, teachers, food service employees, and parents played an important role in program development to ensure the program would be compatible with the needs of the people who would implement it. From 1991 through 1994, researchers funded by the National Heart, Lung, and Blood Institute conducted a 3-year randomized controlled trial evaluating the program in California, Louisiana, Minnesota, and Texas. The evaluation involved ethnically and racially diverse groups of students from 96 elementary schools (7).

The CATCH program is focused on changing the behavior of elementary school students — both in and out of school. Classroom teachers use a prepared, age-appropriate curriculum to teach about physical activity and healthy eating. Students in regular classes practice new skills designed to improve their physical activity and eating behaviors. In physical education classes, teachers encourage students to actively participate in physical activity that is fun. School cafeterias serve healthy, low-fat foods that have been tested for their appeal to elementary students. Study results showed that as a result of the CATCH program, students in the intervention schools significantly increased time spent in moderate to vigorous physical activity within physical education classes (from 40% to 50%) and considerably  decreased their consumption of fat in school meals (from 39% to 32%) (7).

A 3-year follow-up study between 1995 and 1998 assessed diet, physical activity, and related health indicators among 3714 (73%) of the initial CATCH participants. Students receiving the CATCH intervention in grades three through five maintained a diet considerably lower in total fat and saturated fat and participated in more vigorous physical activities in grades six through eight than did students in control groups (8). These findings reinforced the expectation that participation in the CATCH program could contribute to better cardiovascular health as students mature.

A recent replication study of CATCH in a Hispanic community on the U.S.–Mexico border in El Paso, Tex, showed that participation in the CATCH program stemmed the rate of increase in overweight among both girls and boys in grades three through five. Without the CATCH intervention, overweight increased among students in these grades from 26% to 39% among girls and from 40% to 49% among boys (9). These figures can be compared with 30% to 32% for girls and 40% to 41% for boys who participated in CATCH. Thus, participation in the CATCH program appears to help address the obesity epidemic affecting children today, and researchers using the results of the El Paso study found CATCH to be cost-effective (S. Brown, oral communication, June 2005).

Since completion of these two studies, the CATCH program 1) has expanded its  curricula to include kindergarten, first, and second grades; 2) has developed and evaluated an after-school program for kindergarten through fifth grade (10); and 3) has created a physical education module for grades six through eight.

In 1996, with support from the Texas Department of State Health Services, local Texas foundations, and the CDC PRC program, a dissemination team consisting of Texas faculty investigators and field staff responsible for the randomized controlled trial was formed to translate CATCH research into practice. The models eventually selected for dissemination were based on elements from Rogers' diffusion theory (11), social cognitive theory (12), and social marketing (13) as described by Hoelscher et al (14). Factors that influenced the extent of dissemination and adoption included the attributes of the interventions, characteristics of the adopter, types of decisions required to implement the program, channels of communication, social context of the system into which the innovation is introduced, and the efforts of change agents.

The CATCH dissemination team developed strategies to reach the maximum number of school opinion leaders, teachers, and administrators in Texas. By mid-2006, more than 2000 elementary schools in Texas had purchased the CATCH program, and more than 900,000 students had potentially been exposed to the program. Results of mailed surveys to these schools indicated a high level of program implementation and fidelity (15). A recent study showed that training of school staff is important and influences whether or not schools continue to use the CATCH program (16).

**Lessons learned from the CATCH program**

1. During development of the CATCH program, those who were going to use the program (e.g., teachers, food service employees, administrative staff, students, parents) were included in the planning and design. Their inclusion was essential to ensure program acceptability.
2. Successful diffusion requires program staff who can contact decision makers, answer questions, present the program at professional meetings, conduct training, and ensure quality control. 
3. The supportive involvement of a school principal or other administrator is crucial.
4. Effective training of interdisciplinary teams (i.e., classroom and physical education teachers and food service staff) to implement the CATCH program curriculum is important for program success.
5. To gain the support and enthusiastic participation of teachers, prepared lessons aligned with state education standards should be provided along with sufficient training and flexibility in delivering materials.

## Planet Health

Planet Health was developed by researchers at Harvard University in collaboration with teachers and principals (17) for public middle schools. The focus of this interdisciplinary curriculum is to improve cardiovascular health and lower the prevalence of obesity among sixth- through eighth-grade students. The curriculum was designed 1) to fit easily into existing language, mathematics, science, social studies, and physical education classes; 2) to foster basic educational competencies required by the state; and 3) to provide teaching materials that are easy to use. Planet Health's goals for youths are based on evidence that healthy eating and physical activity are important and emphasize increasing consumption of fruits and vegetables and decreasing consumption of high-fat foods.

One Planet Health innovation is promoting child health by stressing the importance of decreasing television viewing. This intervention goal was based on recent evidence that television viewing contributes to obesity through both the sedentary act of television watching and exposure of viewers to relentless broadcast encouragement to consume high-calorie, high-fat, nutrient-poor foods (18,19).

A 21-month randomized controlled study of Planet Health between 1995 and 1997 was funded by the National Institute of Child Health and Human Development in 10 public middle schools. This study showed 1) a reduction in television watching for both girls and boys enrolled in Planet Health compared with controls, 2) a significant decrease in prevalence of obesity among girls in the study, 3) an increase in fruit and vegetable consumption among girl participants, and 4) less of an increase in daily calories consumed by girls enrolled in Planet Health (20). Additional analyses of the data funded by CDC showed that girls participating in Planet Health were less likely to report weight control behavior disorders (21) or early menarche (22). 

CDC researchers conducted an independent economic analysis of Planet Health based on estimated program costs of $14 per student per year and found the program to be highly cost-effective: for every dollar spent on middle school Planet Health programs, researchers projected a savings of $1.20 in medical costs and lost wages by the time students reach middle age (40 to 65 years of age) (23).

Boston Public Schools (BPS) expressed interest in the Planet Health program after they heard about its success from Harvard PRC researchers. A partnership was formed between BPS and the Harvard PRC that included the original Planet Health researchers. This team planned a pilot test of the Planet Health curriculum in BPS public school settings to determine the feasibility and sustainability of the Planet Health program in settings where resources are severely limited.

A BPS and Harvard PRC advisory board provided guidance by using a model of community-based participatory research. BPS selected a sample of six inner-city middle schools to participate, and the PRC provided copies of the Planet Health curriculum, training workshops for teachers, small stipends for teacher coordinators, and evaluation and research expertise. This study was funded by CDC and demonstrated that 90% of the teachers found the curriculum effective and believed it made a positive contribution to their classes (24).

BPS has endeavored to sustain and expand use of the Planet Health curriculum through independent funding. BPS first secured funding from the U.S. Department of Education's Physical Education for Progress grant program for pilot expansion to 12 schools during 2002 through 2003. BPS later received financial support for further expansion from the Boston Public Health Commission under the auspices of the Steps to a HealthierUS (STEPS) project sponsored by the U.S. Department of Health and Human Services. In 2004, BlueCross BlueShield of Massachusetts announced a 4-year, $3-million program of grants to Massachusetts middle schools to implement Planet Health and offer after-school programs. Hundreds of Massachusetts middle school teachers and school administrators have adopted Planet Health, and efforts to disseminate and evaluate the Planet Health curriculum in additional state school systems are under way.

More than 4000 copies of the Planet Health curriculum have been purchased in 48 states and 20 countries. The program has the potential to benefit thousands of children, has demonstrated that it is effective, feasible, and sustainable in a public school environment, and has shown that it is accepted by participants and teachers. In the long term, the program is cost effective and is projected to save society money.

**Lessons learned from Planet Health**

1. Honoring the scientific evidence that television viewing has a major influence on obesity in children was worthwhile. Planet Health included limitation of television viewing among its targets for behavioral change, achieved a considerable reduction, and found this reduction to be beneficial.
2. By working closely with teachers and principals, holding focus groups with students and parents, and working with project advisory boards, Planet Health developed materials that resonated well with the interests and resources of the stakeholders. Participation of all parties was crucial to developing materials that were liked by everyone. For example, researchers would not have known in advance that teachers preferred development of a textbook for their use rather than Web-based materials.
3. From the beginning, researchers looked to the work of Rogers (11) when planning both the diffusion and sustainability of the intervention materials. Researchers were well aware that school systems throughout the United States were reducing their focus on health education and physical education because of financial constraints and the rising importance of high-stakes testing. This understanding led them to develop a low-cost interdisciplinary curriculum that used existing teachers and stressed literacy across the curriculum. These curriculum characteristics tended to increase the perceived relative advantage of the intervention.

## Not-On-Tobacco (N-O-T)

West Virginia led the nation in teenage smoking during the mid–1990s, and as a result, the West Virginia University PRC partnered with West Virginia's Bureau for Public Health, Department of Education, and other members of the state's public health community to strengthen school-based tobacco control initiatives. West Virginia needed an effective, user–friendly teenage smoking cessation program that could be adopted statewide and that would support a newly developed state tobacco-free school policy emphasizing prevention and cessation support rather than punitive action (25,26).

Later inclusion of the American Lung Association (ALA) into the partnership brought a national scope to the West Virginia teenage smoking cessation efforts. The partnership identified local and national needs and took on the shared goal of developing a theoretically based, scientifically tested teenage smoking cessation intervention (26). With funding from CDC and other organizations, the West Virginia University PRC launched a smoking cessation project that was committed to community-based participatory research methods and included the following contributions: 1) teachers, students, and school health professionals provided input for program development; 2) the ALA provided program expertise, funding, and a means for disseminating programs; and 3) PRC researchers provided a scientific framework to evaluate the effectiveness of programs and a commitment to program dissemination.

The partnership decided the intervention should be initially developed for use in schools because schools can promote tobacco-free environments through policy, can offer services for students and staff who want to quit smoking, and can reach youth efficiently (27). Program goals were to create a smoking cessation program that could 1) enhance adolescent health, 2) fulfill the needs of students who want to quit smoking, 3) reduce school tobacco policy violations, and 4) provide an educational alternative to punitive measures for violating school tobacco policy.

Through an iterative, collaborative process, the partnership developed a smoking cessation program designed for 14- to 19-year–old daily smokers (27). The program was given the youth-approved name, Not-On-Tobacco. In addition to smoking cessation, other N-O-T goals include reducing smoking; increasing healthy lifestyle behaviors (e.g., physical activity, healthy eating); and improving stress management, decision making, coping ability, and social support skills. Students participate in the program on a voluntary basis, and the program includes 10 hour-long weekly sessions and 4 booster sessions in same-sex groups with same-sex facilitators (e.g., teachers, school nurses, counselors, volunteers).

Facilitators are trained by the ALA and may lead sessions in both schools and other community settings. They assist participants with 1) identifying reasons for smoking and excuses for not quitting, beliefs and behaviors that reinforce smoking and self-defeating behaviors, triggers for smoking, and other barriers to the quitting process; 2) recognizing and understanding the process of nicotine addiction, advertising ploys to encourage youth smoking, and situations that may spark relapse; and 3) developing skills in cognitive restructuring, coping with stress and peer pressure, identifying and maintaining social supports, goal setting, and assertiveness and other behavior changes (27,28).

After the N-O-T program was developed, partners collaborated to implement the program and assess its efficacy and acceptability in school settings (29-32). Because the need for a youth smoking cessation program was so great, ALA field workers were anxious to use N-O-T even before effectiveness studies were complete. As a result, while the N-O-T demonstration studies in West Virginia and several other states were under way, other local uses of the program were allowed if results were carefully monitored (26).

Over a 7–year period the N-O-T program went through several iterations, testing, refinement, and retesting. Demonstration studies were conducted in West Virginia, North Carolina, Florida, New Jersey, Illinois, Wisconsin, and Washington by the West Virginia team and independent investigators. Studies have consistently shown that adolescents enrolled in N-O-T programs have significantly greater quit and reduction rates than adolescents in more conventional smoking cessation programs. A recent review of N-O-T program outcomes in different locations included more than 6000 youths in 489 schools and showed that after 3 months, adolescents enrolled in N-O-T programs were twice as likely to have quit smoking than adolescents in conventional smoking cessation programs (33). The N-O-T program has been designated a model program by the federal Substance Abuse and Mental Health Services Administration and the federal Office of Juvenile Justice and Delinquency Prevention and is considered a research tested intervention program by the National Cancer Institute.

PRC researchers and the ALA developed a national training prototype by using the tested curriculum and scientific study results and initiated a N-O-T media campaign in 2000. The ultimate success of an evidence-based program is reflected in its widespread dissemination and adoption (34), and as of 2005, N-O-T trainings have occurred in 47 states and in Europe and Canada. It is estimated that since 1998, at least 100,000 adolescents have participated in N-O-T programs in the United States (American Lung Association, 2005). N-O-T program leaders estimate conservatively that one in five participants quits smoking (33); this rate suggests that N-O-T programs have helped up to 20,000 U.S. adolescents quit smoking. In West Virginia, there are trained N-O-T facilitators in 75% of high schools, and program dissemination is supported by the Bureau for Public Health, the Department of Education, and the American Lung Association's West Virginia chapter.

N–O–T programs are supported in Canada and a number of U.S. states. Some states, including Virginia, Vermont, Wisconsin, Delaware, and Rhode Island, incorporate the N-O-T program into their state tobacco control plans.

**Lessons learned from the N-O-T program**

1. Involvement of multiple stakeholders, including school personnel and students, in the N-O-T program design resulted in a program that is feasible and effective and that attracts local champions to spearhead implementation in multiple locales.
2. Dissemination of the N-O-T program was a goal from its beginning, and this aim was a valuable guide to keep the program practitioner-friendly, consistent with local policies, and appealing to local funding agencies.
3. Having a partner with experience in national dissemination (the ALA) provided the capacity for widespread diffusion and adoption of the N-O-T program.

## Discussion

The lessons learned in the course of developing and disseminating these three school-based programs — CATCH, Planet Health, and N-O-T — are remarkably similar. Each program included multiple stakeholders to plan, implement, evaluate, and disseminate interventions. Although stakeholder groups varied in some ways, school administrators and teachers were included in all three programs. PRC researchers became familiar with the culture of schools and learned that a school-based intervention must be easy and inexpensive to implement, needs to allow flexibility for local adaptation, and should be aligned with current political constraints and academic mandates to be successful. All three programs provided teacher-friendly, inexpensive prepared curricula that appealed to students. Training was crucial to all three programs but was not burdensome in terms of time commitment or budget. Planning for dissemination from the start helped maintain a focus on feasibility and acceptability.

All three programs used the work of Rogers (11) as a foundation for design and dissemination efforts. His diffusion theory posits that adoption is more likely when innovations are perceived to have 1) relative advantage (the innovation is better than the status quo), 2) compatibility (the innovation is consistent with current values), 3) low complexity (the innovation is not difficult to understand and use), 4) observability (results of the innovation are noticeable to others), and 5) trialability (the innovation can be tried out on a partial or temporary basis). Other factors that encourage adoption, use, and maintenance of programs are involvement of advocates and adequate training to enhance user competency.

Another characteristic common to these programs was that each was shown to be effective through sound research studies. There is no substitute for demonstrating through methodologically sound science that an intervention leads to desirable behavior change (when compared with the status quo) in real-world settings. Such evidence of effectiveness also helps promote sustainable support since external agencies that fund school health programs are likely to favor those that are shown to be effective. Each of these programs has found strong local interest and financial support.

PRC involvement is an important feature shared by these three successful school-based programs. The CDC-administered PRC program is intended to build durable partnerships between public health professionals and organizations, academia, schools, community organizations, and community members to foster development and dissemination of programs that improve community health. Improving and maintaining community health requires a stable infrastructure that allows cultivation and maintenance of complex partnerships. PRC funding helps create this  necessary infrastructure by supporting staff dedicated to improving health in underserved communities and by providing scientific research and evaluation expertise. PRCs help hold partnerships together and make it possible for local institutions to seek sustainable funding for effective health programs and polices.

The lessons learned from these three programs should encourage public health practitioners to consider schools as a potential venue for public health improvement. Public health practitioners who wish to work with schools, whether to implement programs or conduct research, need to develop an understanding of the school environment and how educational bureaucracy is structured (35). Schools are best approached through appropriate channels, such as a Coordinated School Health Program, a state or district coordinator, or an individual school health coordinator. These sources understand the current school policy environment in the state and can provide pertinent information, advice, and routes of communication.

Public health professionals can engage schools on their own ground to demonstrate the value of public health to school personnel. By attending local school board or district school health council meetings, public health professionals can offer valuable information to develop policies to promote high-quality health education and physical education courses, high-quality school food services, and a healthy school environment. Public health practitioners can also work with state coalitions for healthy schools or participate in professional organizations with a mission to support school health programs. Resources to build school health programs, such as promising practices for state leaders, funding opportunities, evidence-based guidelines, key strategies, and physical education curriculum analysis tools are available at www.cdc.gov/healthyyouth.

The School Health Index (SHI) is a CDC resource that can assist public health partnerships with schools through a structured process that helps schools assess their competencies in components of a coordinated school health program. The SHI requires interdisciplinary involvement from school personnel, promotes a collaborative culture within participating schools, and raises awareness of needs for health-related curricula, services, and policies. Schools that have used SHI programs are primed to improve their overall school health program, to welcome the involvement of public health experts who offer to test new health promotion programs, and to consider implementation of programs shown to be effective elsewhere.

Although challenges to working with schools are substantial, experience shows that success is possible. Understanding the school milieu, ensuring participation of relevant stakeholders (e.g., teachers, administrators, students, parents) in all stages, keeping potential dissemination in mind in all stages, and providing ample training and easy-to-use prepared curricula are features of the successful school-based interventions promoted by the PRC program.

## Appendix

The following Web sites are for organization and agency information used in research for this article.

1. **Action for Healthy Kids.**
  http://www.actionforhealthykids.org/
2. **American Lung Association Not On Tobacco Program.**
  http://www.lungusa.org/site/pp.asp?c=dvLUK9O0E&b=39866
3. **American School Health Association.**
  http://www.ashaweb.org/
4. **CATCH Texas.**
  http://www.sph.uth.tmc.edu/catch/
5. **CDC Healthy Youth Program School Health Index.**
  http://apps.nccd.cdc.gov/shi/default.aspx
6. **Harvard Prevention Research Center on Nutrition and Physical Activity, Planet Health.**
  http://www.hsph.harvard.edu/prc/proj_planet.html
7. **National Association of State Boards of Education (NASBE) Center for Safe and Healthy Schools.**
  http://www.nasbe.org/healthy_schools/intro.htm
8. **National Cancer Institute Research–tested Intervention Programs.**
  http://rtips.cancer.gov/rtips/index.do
9. **Office of Juvenile Justice and Delinquency Prevention, U.S. Department of Justice.**
  http://www.ojjdp.ncjrs.org/programs/mpg.html
10. **Substance Abuse and Mental Health Services Administration (SAMHSA) Model Programs.**
  http://www.modelprograms.samhsa.gov/template_cf.cfm?page=model&pkProgramID=521
11. **Rhode Island Tobacco Control Program School Tobacco Cessation.**
  http://www.health.state.ri.us/disease/tobacco/school-cessation.php
